# Assembly of Hydrophobic ZIF-8 on CeO_2_ Nanorods as High-Efficiency Catalyst for Electrocatalytic Nitrogen Reduction Reaction

**DOI:** 10.3390/nano12172964

**Published:** 2022-08-27

**Authors:** Yiwen Liu, Xianbin Meng, Zhiqiang Zhao, Kai Li, Yuqing Lin

**Affiliations:** Department of Chemistry, Capital Normal University, Beijing 100048, China

**Keywords:** electrocatalytic nitrogen reduction reaction, hydrophobicity, assembly strategy, CeO_2_-ZIF-8

## Abstract

The electrocatalytic nitrogen reduction reaction (NRR) can use renewable electricity to convert water and N_2_ into NH_3_ under normal temperature and pressure conditions. However, due to the competitiveness of the hydrogen evolution reaction (HER), the ammonia production rate (R_NH__3_) and Faraday efficiency (FE) of NRR catalysts cannot meet the needs of large-scale industrialization. Herein, by assembling hydrophobic ZIF-8 on a cerium oxide (CeO_2_) nanorod, we designed an excellent electrocatalyst CeO_2_-ZIF-8 with intrinsic NRR activity. The hydrophobic ZIF-8 surface was conducive to the efficient three-phase contact point of N_2_ (gas), CeO_2_ (solid) and electrolyte (liquid). Therefore, N_2_ is concentrated and H^+^ is deconcentrated on the CeO_2_-ZIF-8 electrocatalyst surface, which improves NRR and suppresses HER and finally CeO_2_-ZIF-8 exhibits excellent NRR performance with an R_NH__3_ of 2.12 μg h^−1^ cm^−2^ and FE of 8.41% at −0.50 V (vs. RHE). It is worth noting that CeO_2_-ZIF-8 showed excellent stability in the six-cycle test, and the R_NH__3_ and FE variation were negligible. This study paves a route for inhibiting the competitive reaction to improve the NRR catalyst activity and may provide a new strategy for NRR catalyst design.

## 1. Introduction

As a potential alternative to Haber–Bosch, electrocatalytic nitrogen reduction reaction (NRR) is a green and sustainable method to produce NH_3_ [1,2,3,4,5,6,7,8,9,10,11]. As we all know, the electrocatalytic NRR process, as opposed to the simple two-electron reaction mechanism of hydrogen evolution reaction (HER), includes numerous multiphase reactions involving six protons, six electrons, and one N_2_ and a complicated mass transfer process [8]. A non-negligible fact is that the extremely low solubility of N_2_ in electrolyte limits the supply of N_2_ molecules to the NRR process, while protons (H) in aqueous solution are easily dissociated in water, resulting in HER with overwhelming competition [7]. In addition, studies have shown that most catalytic materials are intrinsically favorable for the adsorption of H atoms rather than N_2_ molecules, resulting in the majority of surface-active centers and electrons being occupied by undesirable H atoms, which then end up at poor selectivity [7,12,13,14,15,16]. From the perspective of thermodynamics, although both NRR and HER need similar theoretical potentials, due to the strong dipole moment and the ultrahigh bond energy of the strong N≡N triple bond, with a bond energy of 940.95 kJ mol^−1^, NRR can only proceed at a higher overpotential than HER [17]. As a result, in the potential window of an electrocatalytic NRR process, undesirable HER processes often predominate and severely lower the ammonia production rate (R_NH_3__) and Faraday efficiency (FE) of NRR.

In the face of this critical challenge, one viable strategy is to increase the concentration of N_2_ on the catalyst surface in order to boost NRR and inhibit HER. Gas-phase electrochemical reactions have been greatly influenced by hydrophobic interfaces in recent years since they can provide rich three-phase contact points (TPCPs) for gas, catalyst and electrolyte [18]. In addition, the hydrophobic interface offers a quick channel for gas diffusion, supplying the catalyst surface with a sufficient amount of gas [19,20,21,22]. Therefore, instead of being wetted by electrolyte, the electrochemical gas evolution process is more likely to occur on the hydrophobic surface of the TPCPs [19]. Additionally, H^+^ concentration at the hydrophobic interface is lower than that at the hydrophilic interface since there is inadequate contact between the aqueous and the hydrophobic surface [20,21,22,23]. Therefore, it stands to reason that designing a three-phase electrocatalyst with hydrophobic interfaces would be a successful way to increase NRR and suppress HER. Du and Ling et al. introduced a hydrophobic zeolite imidazolate framework (ZIF) to cover Au Ag-Au or Pt/Au by the surface modification of electrocatalyst, forming a three-phase interface that inhibits HER and enhances electrochemical NRR [24,25,26]. However, these strategies involved precious metals which are scarce and expensive. Cerium oxide (CeO_2_) rich in oxygen vacancies has been reported to possess intrinsic NRR activity since the flexible conversion between +3 and +4 valence in CeO_2_ offers the coordinatively unsaturated sites for electron transfer to the adsorbed N_2_ molecule and weakens the nitrogen–nitrogen bond [7,27,28,29]. The N≡N triple bond can be softened for future activation and hydrogenation by injecting the abundant oxygen vacancy in CeO_2_ into the antibonding orbital of N_2_ adsorbed on the surface of the catalyst [7,27,28,29]. Related research provides a basis for the subsequent research and development of CeO_2_-based electrocatalytic NRR catalysts.

The metal–organic frameworks (MOFs) are an emerging porous crystalline materials, among which the ZIFs are a broad sub-category and have great applications in the chemical catalysis of various reactions [30,31]. However, due to the few active sites and low conductivity, the electrocatalytic activity of ZIFs is usually poor [32]. However, ZIFs can be used as a matrix or dopant to immobilize other active electrocatalysts and improve certain properties [25,33]. Firstly, their porosity promotes chemical accumulation around the active sites and reduces diffusion in the electrolyte [25,32]. Secondly, the strong chemical endurance of ZIFs preserves the catalyst’s structural integrity. In addition, some ZIFs (such as ZIF-8) show good hydrophobicity, so they can effectively inhibit HER [25,26]. The porosity and hydrophobicity of ZIF-8 are favorable for the enrichment of N_2_ near the active site. Furthermore, ZIF-8 is stable in electrolyte, which can enhance the overall cycling performance of the catalyst.

**Scheme 1:**
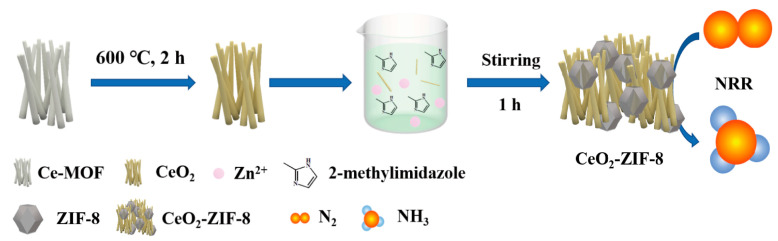
Synthetic processes of CeO_2_-ZIF-8.

In our previous work, the synthesized CeO_2_ with rich oxygen vacancies proved to have intrinsic NRR activity [27]. In this work, the co-assembled electrocatalytic nitrogen reduction catalyst CeO_2_-ZIF-8 was synthesized by incorporating porous hydrophobic ZIF-8 on CeO_2_ nanorods with abundant oxygen vacancies (Scheme 1). The experimental demonstration showed that, by improving the hydrophobicity of the catalyst, the progress of HER during the electrocatalytic nitrogen reduction process was inhibited and the enrichment of N_2_ near the active site was enhanced. Meanwhile, the dodecahedron structure of ZIF-8 can promote the uniform distribution of CeO_2_, prevent CeO_2_ from agglomerating, and expose more active sites in CeO_2_, thus improving the NRR catalytic activity with R_NH_3__ of 2.12 μg h^−1^ cm^−2^ and FE of 8.41% at −0.50 V (vs. RHE). The research provides ideas for inhibiting the competitive reaction HER by increasing the hydrophobicity, and then promoting NRR.

## 2. Experimental Section

### 2.1. Synthesis of CeO_2_

In a classical process [34], 1,3,5-Benzenetricarboxylic acid (1 mmol) and Cerium (III) nitrate hexahydrate (1 mmol) were added to 50 mL of the combined solution (water-to-ethanol ratio was 1:1), and kept under stirring conditions constantly at room temperature. Then, the solution was heated to 90 °C for two hours to obtain Ce-MOF. The resulting Ce-MOF (white powder) was then dried at 70 °C and centrifuged after being cleaned six times with water and ethanol. The Ce-MOF is then heated to 600 °C for two hours with a 5 °C/min temperature rise in air to produce pale yellow CeO_2_.

### 2.2. Synthesis of CeO_2_-ZIF-8

To prepare CeO_2_-ZIF-8, 55 mg of CeO_2_, 148 mg Zn(NO_3_)_2_·6H_2_O, 2-methylimidazole were homo-dispersed in 20 mL of methanol, and the mixture solution was stirred for 3 h. The end product, together with the precipitates on the bottom, was carefully collected, washed five times in ethanol, and then dried for ten hours at 70 °C.

### 2.3. Synthesis of CeO_2_-ZIF-8 on Carbon Paper (CPs)

The CPs (1 cm × 1 cm × 0.1 cm) were submerged in a 70% concentrated HNO_3_ solution at 115 °C for 1.5 h, rinsed three times with water thereafter, and dried at 65 °C before being used. Then, 1 mg CeO_2_-ZIF-8 powder was dispersed in a mixture of 50 μL water, 50 μL ethanol and 5 μL 5 wt% Nafion ethanol. After ultrasonic treatment for 30 min, the dispersion was evenly dropped onto CPs.

## 3. Results and Discussion

### 3.1. Investigation of Morphology and Structure of CeO_2_-ZIF-8

**Figure 1:**
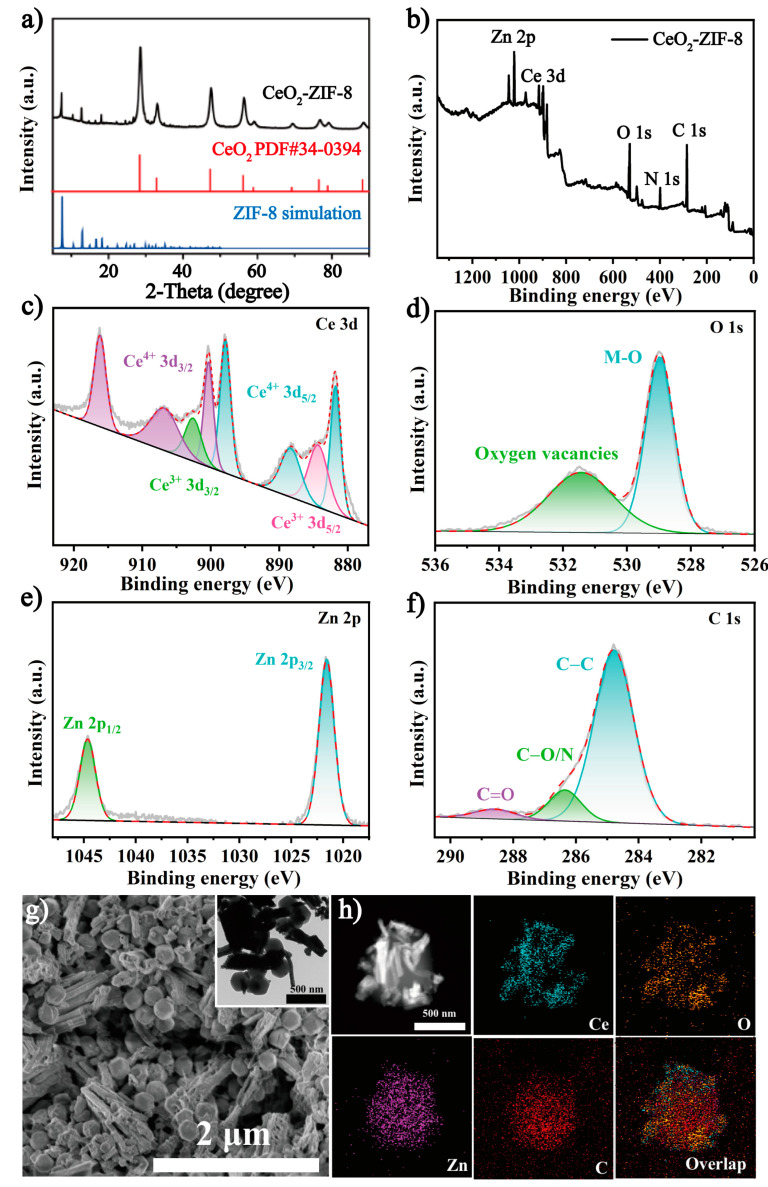
(**a**) XRD pattern and (**b**) full XPS spectrum of CeO_2_-ZIF-8. (**c**–**f**) constitutional element XPS spectra of Ce 3d, O 1s, Zn 2p, and C 1s. (**g**) SEM image and TEM image (inset) of CeO_2_-ZIF-8. (**h**) Mapping images of CeO_2_-ZIF-8.

To learn more about the crystalline structure of CeO_2_-ZIF-8, the XRD method was used (Figure 1a). The CeO_2_ crystal planes (PDF#34-0394) (111), (200), (220), and (311) may be readily connected with the four strong diffraction peaks centered at 2θ = 28.6°, 33.1°, 47.5°, and 56.3°. Meanwhile, a series of diffraction peaks dominated by 2θ = 7.4°, 12.8° and 18.1° are consistent with the simulated XRD pattern of ZIF-8, indicating that ZIF-8 can exist stably in CeO_2_-ZIF-8. X-ray photoelectron spectroscopy (XPS) was used to analyze the types and valence states of the metal elements on the surface of the synthesized sample. As shown in Figure 1b, the full XPS spectrum of CeO_2_-ZIF-8 shows that the catalyst has the element of Ce, O, Zn, C, N. The Ce 3d peak (Figure 1c) in CeO_2_-ZIF-8 can be divided into eight characteristic peaks. The peaks at 881.78 eV, 888.32 eV and 897.88 eV correspond to the characteristic peak of Ce^4+^ 3d 5/2 (in blue), and the peaks at 900.32 eV, 902.58 eV and 916.18 eV correspond to the characteristic peak of Ce^4+^ 3d 3/2 (in purple). The peaks at 884.32 eV and 902.58 eV correspond to the characteristic peaks of Ce^3+^ 3d 5/2 (in pink) and Ce^3+^ 3d 3/2 (in green), respectively [7,27,35,36,37]. In CeO_2_, Ce exists in +3 and +4 valences. This difference in the number of valence states can lead to the changes in the amount of oxygen vacancies in CeO_2_, which helps to improve the adsorption of N_2_ by the catalyst, and the subsequent dissociation and hydrogenation of N≡N, thereby showing a certain NRR activity. The O 1s XPS spectrum (Figure 1d) also verify the oxygen vacancies with the peak at 531.40 eV (in green), and the peak at 529.00 eV (in blue) which indicates the metal–oxygen band (M-O) [7]. The XPS data of CeO_2_-ZIF-8 and CeO_2_ were compared. Among them, the Ce 3d and O 1s spectra in CeO_2_-ZIF-8 shifted to the lower energy level by 1.0 eV and 0.7 eV, respectively, indicating that before and after the co-assembly of CeO_2_ and ZIF-8, ZIF-8 may affect the electron binding energies of the Ce and O elements, inducing them shifting to lower energy levels, which may facilitate the transfer of electrons from the catalyst to the reactants (Figure S1). The Zn 2p peak (Figure 1e) in CeO_2_-ZIF-8 can be divided into Zn 2p 3/2 at 1043.73 eV (in blue) and Zn 2p 1/2 at 1020.71 eV (in green). For the three peaks in the C 1s XPS spectrum (Figure 1f) at 288.56 eV (in purple), 286.40 eV (in green) and 284.80 eV (in blue) correspond to C=O, C-O/N and C-C bonds, respectively.

The morphology of CeO_2_-ZIF-8 samples was characterized by SEM and TEM (inset), as shown in Figure 1g. It can be seen that the synthesized CeO_2_-ZIF-8 is a co-assembly mixture of nanorods and dodecahedron, where the nanorods are CeO_2_ and the dodecahedron are ZIF-8. As shown in Figure S2, individual CeO_2_ nanorods have a tendency to agglomerate, which prevents the full exposure of active sites in CeO_2_ and affects the further improvement of NRR activity. In comparison to CeO_2_ alone (as shown in Figure S2), the co-assembly CeO_2_-ZIF-8 (as shown in Figure S3) shows that CeO_2_ and ZIF-8 are evenly distributed, and the porosity and hydrophobicity of ZIF-8 are advantageous for the enrichment of N_2_ at the active sites, hence boosting NRR activity [25,26]. In addition, the element mapping imaging technology was also applied to obtain CeO_2_-ZIF-8 element distribution and topography information. As shown in Figure 1h, the Ce, O, Zn and C elements of CeO_2_-ZIF-8 are uniformly distributed in the sample, which proves that the co-assembly structure of CeO_2_-ZIF-8 has a uniform distribution for both CeO_2_ and ZIF-8, which is beneficial to the uniform distribution and full exposure of the catalytic sites.

**Figure 2:**
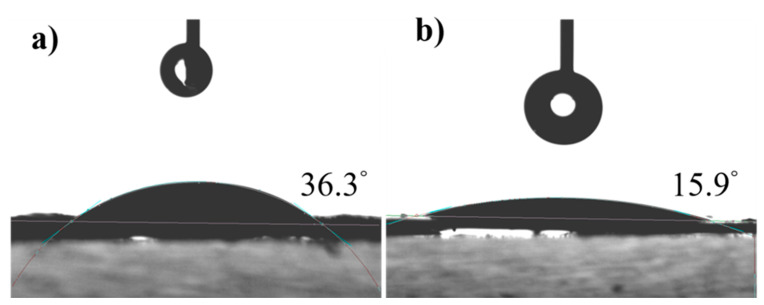
Water contact angle measurement of (**a**) CeO_2_-ZIF-8 and (**b**) CeO_2_ substrates.

Increasing the hydrophobicity can inhibit the HER in the NRR reaction process, which helps to improve the selectivity of the catalyst. In order to explore the hydrophobicity of CeO_2_-ZIF-8, the contact angle of the samples CeO_2_-ZIF-8 and CeO_2_ was tested. The 0.5 M K_2_SO_4_ aqueous solution contact angle on CeO_2_-ZIF-8 is 36.3°, as shown in Figure 2a, which is an increase of 20.4° over the 15.9° on CeO_2_ shown in Figure 2b. The results show that, after CeO_2_ is combined with ZIF-8 to create a CeO_2_-ZIF-8 co-assembly structure, the contact angle of the catalyst material has been significantly increased, which proves that its hydrophobicity has increased. In the process of nitrogen reduction, CeO_2_ acts as a catalyst. The porous hydrophobic ZIF-8 co-assembled with the active site improved the hydrophobicity of the catalyst and the enrichment degree of nitrogen on the catalyst surface and inhibited HER, which in turn can promote the catalytic activity of NRR [24,25].

### 3.2. Electrocatalytic Nitrogen Reduction Performance

**Figure 3:**
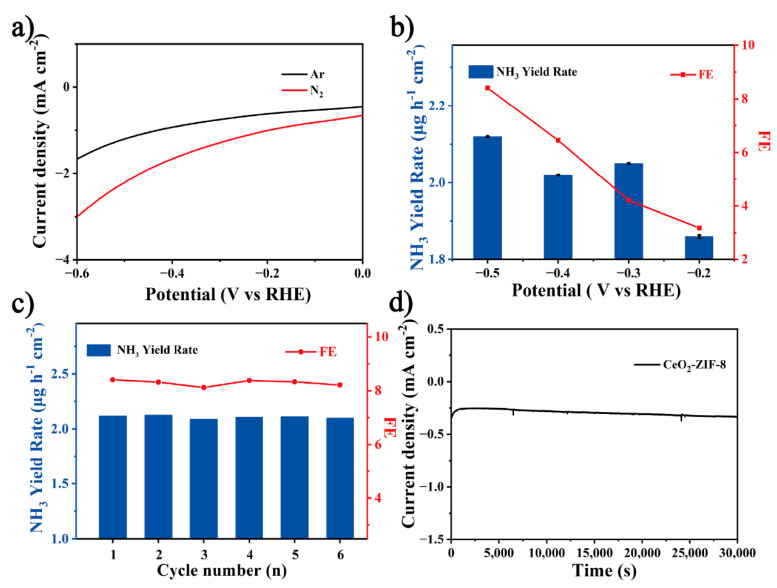
(**a**) NRR electrochemical performances of CeO_2_-ZIF-8 in 0.5 M K_2_SO_4_ at a scan rate of 2 mV/s; (**b**) Faradaic efficiency (red) and yield rate of NH_3_ (blue) at each potential; (**c**) cycling test of the CeO_2_-ZIF-8 at −0.5 V vs. RHE for 2 h of each NRR experiment in N_2_-saturated 0.5 M K_2_SO_4_ solution (pH 3.5); (**d**) time-dependent current density during electrolysis at −0.5 V vs. RHE for 30,000 s.

The NRR activity of the CeO_2_-ZIF-8 was first measured by linear sweep voltammetry (LSV) in a 0.5 M K_2_SO_4_ solution saturated with Ar and N_2_, respectively, as shown in Figure 3a. Compared with Ar saturation, the current density of CeO_2_-ZIF-8 significantly increases under N_2_ saturation, which proves that nitrogen is involved in the cathodic reduction reaction, which means that CeO_2_-ZIF-8 has electrocatalytic activity towards NRR. In order to verify and quantify the product of nitrogen reduction, the indophenol blue method was introduced to detect the output of ammonia in the electrolyte. The standard concentration of ammonia solution was prepared in 0.5 M K_2_SO_4_ aqueous solution, and the standard concentration curve of ammonia solution was plotted by UV–Vis absorption spectrum through the indophenol blue method. The calibration curve (y = 0.638x + 0.209, R^2^ = 0.999) shows an excellent linear correlation between concentration and absorbance by three independent calibrations (Figure S4). For quantitatively detecting the R_NH_3__ and FE at various potentials, chronoamperometry test was introduced, and the indophenol blue method was utilized to measure the ammonia content of the electrolyte by UV–Vis (Figure S5) [38]. As shown in Figure 3b, the results show that the NRR reaction was already proceeding at −0.2 V, and among the selected four potentials, the working electrode CeO_2_-ZIF-8 loaded with catalyst materials has the highest R_NH_3__ of 2.12 μg h^−1^ cm^−2^ and FE of 8.41% at −0.5 V vs. RHE, which can compete with the bulk of published water-based NRR electrocatalysts, including iron-based catalysts such as the 30%-Fe_2_O_3_-carbon nanotube (0.11 μg h^−1^ cm^−2^), Fe/Fe oxide (0.19 μg h^−1^ cm^−2^), and noble metal catalysts such as Au nanorods (1.65 μg h^−1^ cm^−2^), for which detailed comparative information is included in Table 1 [39,40,41,42,43,44,45]. We carried out two electrolysis procedures at −0.50 V in Ar-saturated and N_2_-saturated solution in order to demonstrate that all of the measured ammonia is a result of the electrocatalytic NRR of CeO_2_-ZIF-8. As illustrated in Figure S6, at an open circuit potential of 0.214 V vs. RHE, the same electrolysis was also performed in an electrolyte that was N_2_-saturated and Ar-saturated at −0.5 V. The corresponding UV–Vis absorption spectrum shows that no ammonia was produced under the conditions of N_2_-saturated at open circuit potential and Ar-saturated at −0.5 V, but ammonia was produced under the conditions of N_2_-saturated at −0.5 V, indicating that all the detected ammonia originated from the NRR reaction on CeO_2_-ZIF-8.

**Figure 4:**
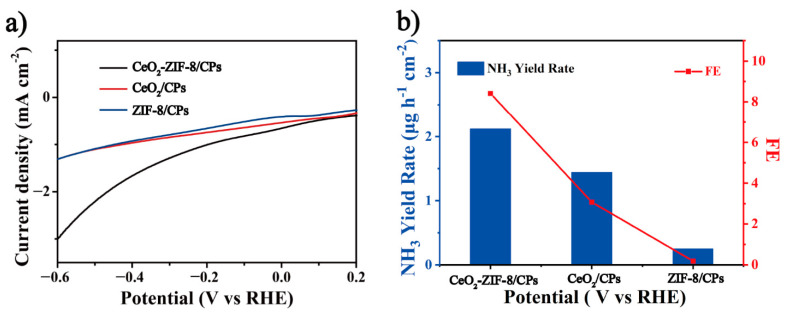
(**a**) The NRR electrochemical performances of CeO_2_-ZIF-8/CPs, CeO_2_/CPs and ZIF-8/CPs in 0.5 M K_2_SO_4_ of nitrogen saturated at a scan rate of 2 mV/s; and (**b**) Faradaic efficiency (red) and yield rate of NH_3_ (blue) of CeO_2_-ZIF-8/CPs, CeO_2_/CPs and ZIF-8/CPs.

Stability is another important indicator for evaluating catalyst performance. In order to determine the stability of the catalyst material, cycle stability and long-term stability tests were introduced. In the catalyst’s cycle stability test (Figure 3c and Figure S7) at −0.5 V vs. RHE, the R_NH_3__ of the first cycle was 2.12 μg h^−1^ cm^−2^ and FE was 8.41%, while the R_NH_3__ of the sixth cycle was 2.10 μg h^−1^ cm^−2^ (99.06% retention rate) and FE was 8.21% (97.62% retention rate), which revealed that the FE and R_NH_3__ did not change significantly. At the same time, the current density did not vary much during the 30,000 s long-term stability test, as shown in Figure 3d. The initial current is basically stable at −0.26 mA cm^−2^, and then the current gradually reaches −0.33 mA cm^−2^ after a longtime test of 30,000 s. This demonstrates the catalyst material’s high stability. The same conclusion can be drawn from the XPS pattern (Figure S8) and SEM (Figure S9) that CeO_2_-ZIF-8 did not change before and after the NRR durability test. All these results show that CeO_2_-ZIF-8 possess high mechanical strength and chemical stability. The high selectivity of CeO_2_-ZIF-8 also confirmed that only NH_3_ but no N_2_H_4_ was formed in the electrolyte. The Watt and Chrisp method was used to verify the quantity of N_2_H_4_ generated in 0.5 M K_2_SO_4_ solutions [46]. The standard concentration of hydrazine solution was prepared in 0.5 M K_2_SO_4_ aqueous solution, and the standard concentration curve of hydrazine solution was drawn by UV–Vis absorption spectrum through the Watt and Chrisp method. The calibration curve (y = 0.738x + 0.307, R^2^ = 0.999) shows an excellent linear correlation between concentration and absorbance by three independent calibrations (Figure S10). The electrolysis procedures were performed for 2 h in 0.5 M K_2_SO_4_ aqueous solution saturated with N_2_ at −0.5 V vs. RHE (Figure S11). Moreover, the Watt and Chrisp method was used to detect the N_2_H_4_ content in the solution before and after the reaction. The results show that the N_2_H_4_ content in the solution before and after the reaction was the same, indicating that there was no hydrazine generation in the electrolysis procedures, which verified the high selectivity of CeO_2_-ZIF-8 for electrocatalytic NRR. In order to explore whether the hydrophobic structure of ZIF-8 can promote the improvement of the NRR performance of the catalyst in the structure of CeO_2_-ZIF-8, the NRR activity of CeO_2_ was also discussed. As shown in Figure 4a, the working electrode loaded with CeO_2_-ZIF-8 has a significantly higher current density than that loaded with CeO_2_ and ZIF-8, indicating that more strong electrochemical reactions occurred on CeO_2_-ZIF-8. As shown in Figure 4b, the results show that the R_NH_3__ and FE of CeO_2_-ZIF-8/CPs are significantly improved compared to CeO_2_/CPs and ZIF-8/CPs. It was proven that the nitrogen reduction performance of ZIF-8 and CeO_2_ catalyst was significantly improved after the co-assembly of the hydrophobic ZIF-8 and CeO_2_. The excellent performance of CeO_2_-ZIF-8 towards NRR catalysis was confirmed by all of these assays.

In summary, the reasons for the excellent NRR performance of CeO_2_-ZIF-8 were as follows: (i) the oxygen vacancies in CeO_2_ offers the coordinatively unsaturated sites for the electron transfer to the adsorbed N_2_ molecule and ensures the intrinsic NRR activity; (ii) the porous hydrophobic ZIF-8 co-assembled with the active site improved the hydrophobicity of the catalyst and the enrichment degree of nitrogen on the catalyst surface and inhibited HER; and (iii) the introduction of ZIF-8 with a framework structure can effectively inhibit the agglomeration of CeO_2_, increase the exposure of catalytic sites, and thereby increase the catalytic activity of NRR.

## 4. Conclusions

In summary, the CeO_2_-ZIF-8 electrocatalytic nitrogen reduction catalyst was prepared by the co-assembly of CeO_2_ nanorods with NRR intrinsic activity and porous hydrophobic ZIF-8. Hydrophobic ZIF-8 can limit the competitive hydrogen evolution reaction HER, allowing more electrons to flow into the electrocatalytic NRR process, endowing CeO_2_-ZIF-8 excellent NRR performance with an R_NH_3__ of 2.12 μg h^−1^ cm^−2^ and FE of 8.41% at −0.50 V (vs. RHE). This study not only increased the reactant concentration but also inhibited the competitive reaction to improve the catalyst activity and current utilization, providing us with a new strategy to improve the activity of the NRR catalyst.

## Figures and Tables

**Table 1:** Comparison of the electrocatalytic N_2_ reduction performance for CeO_2_-ZIF-8/CPs with other aqueous-based NRR electrocatalysts.

Catalyst	Electrolyte	NH_3_ Yield	FE (%)	Ref.
30%-Fe_2_O_3_-CNT	0.50 M KOH	0.11 μg h^−1^ cm^−2^	0.59	[39]
Fe/Fe Oxide	0.10 M PBS	0.19 μg h^−1^ cm^−2^	8.29	[40]
Fe_3_O_4_/Ti	0.10 M Na_2_SO_4_	3.43 μg h^−1^ cm^−2^	2.60	[41]
PEBCD/C	0.50 M Li_2_SO_4_	1.58 μg h^−1^ cm^−2^	2.85	[42]
Fe/Fe Oxide	0.10 M PBS	0.19 μg h^−1^ cm^−2^	8.29	[43]
Ag nanosheets	0.10 M HCl	2.80 μg h^−1^ cm^−2^	4.80	[44]
Au nanorods This work	0.10 M KOH 0.50 M K_2_SO_4_	1.65 μg h^−1^ cm^−2^ 2.21 μg h^−1^ cm^−2^	4.00 8.41	[45]

## Data Availability

The datasets generated during and/or analyzed during the current study are available from the corresponding authors.

## Supplementary Materials

The following supporting information can be downloaded at: https://www.mdpi.com/article/10.3390/nano12172964/s1: Experimental Procedures. Figure S1: Constitutional element XPS spectra of (a) Ce 3d and (b) O 1s of CeO_2_ and CeO_2_-ZIF-8. Figure S2: SEM images of CeO_2_ with different magnification. Figure S3: SEM images of CeO_2_-ZIF-8 with different magnification. Figure S4: Absolute calibration of the indophenol blue method using ammonium chloride solutions of known concentration as standards. (a) UV-Vis curves of indophenol assays with NH^4+^ ions after incubated for 2 h and (b) calibration curve used for estimation of NH_3_ by NH^4+^ ion concentration. The absorbance at 655 nm was measured by UV-Vis spectrophotometer, and the fitting curve shows good linear relation of absorbance with NH^4+^ ion concentration (y = 0.638x + 0.209, R^2^ = 0.999) of three times independent calibration curves. Figure S5: (a) Chronoamperometry results at the corresponding potentials of CeO_2_-ZIF-8/CPs, and (b) UV-Vis absorption spectra of the K_2_SO_4_ electrolyte stained with indophenol indicator after charging at each restricted potential vs. RHE under N_2_ controls for 2 h. Figure S6: UV-Vis absorption spectra of the electrolytes stained with indophenol indicator after 2 h NRR electrolysis under different conditions: open circuit in N_2_ (red curve), −0.5 V in N_2_ (purple curve) and −0.5 V in Ar (blue curve). Figure S7: Cycling test of the CeO_2_-ZIF-8/CPs after consecutive recycling electrolysis in N_2_-saturated 0.5 M K_2_SO_4_ solution (pH 3.5) at −0.5 V vs. RHE for 2 h of each NRR experiment. Figure S8: Full XPS spectrum of CeO_2_-ZIF-8/CPs before (a) and after (b) NRR. Figure S9: SEM images of CeO_2_-ZIF-8/CPs before (a) and after (b) NRR. Figure S10: UV-Vis absorption spectra of various N_2_H_4_ concentrations after incubated for 10 min at room temperature. Calibration curve used for estimation of N_2_H_4_ concentrations. The absorbance at 425 nm was measured by UV-Vis spectrophotometer, and the fitting curve shows good linear relation of absorbance with N_2_H_4_·H2O concentration (y = 0.748x + 0.307, R^2^ = 0.999) of three times independent calibration curves. Figure S11: UV-Vis absorption spectrum of the by-produced N_2_H_4_ for CeO_2_-ZIF-8/CPs tested in 0.5 M K_2_SO_4_ with bubbled N_2_ under −0.5 V vs. RHE for 2 h.
