# Supplementary material for: Assembly of Hydrophobic ZIF-8 on CeO2 Nanorods as High-Efficiency Catalyst for Electrocatalytic Nitrogen Reduction Reaction
Source: Nanomaterials (Basel). 2022 Aug 27;12(17):2964. doi: 10.3390/nano12172964 (PMC9458198; doi:10.3390/nano12172964)
Supplement: Supplementary file 1 [file nanomaterials-12-02964-s001.zip › nanomaterials-1855444-supplementary.pdf]

nanomaterials

Supplementary Material

Assembly of Hydrophobic ZIF-8 on CeO_2 Nanorods as High-Efficiency Catalyst for Electrocatalytic Nitrogen Reduction Reaction

Yiwen Liu †, Xianbin Meng †, Zhiqiang Zhao, Kai Li * and Yuqing Lin *

Department of Chemistry, Capital Normal University, Beijing 100048, China
* Correspondence: likai@cnu.edu.cn (K.L.); linyuqing@cnu.edu.cn (Y.L.)
† These authors contributed equally to this paper.

1. Experimental Procedures

Reagents and Materials: 1,3,5-Benzenetricarboxylic acid (99%) and 2-methylimidazole (98%) were purchased form Johnson & Johnson Chemical Co., LTD. Cerium (III) nitrate hexahydrate (99.5%) and $Zn(NO_3)_2\cdot 6H_2O$ (99.95%) were purchased from Aladdin Re-agent (Shanghai) Co., LTD. Carbon paper (30 cm 30 cm 0.1 cm) was purchased form Toray Corporation of Japan. Methanol, Nafion, ethanol and HNO_3 were purchased from Si-nopharm Chemical Reagent Co. LTD, Shangai, China. The deionized water was purified by the RO-DI system. The purity of the chemicals used in this study were of analytical grade and could be used without further purification.

Characterization: The as-synthesized samples were examined by Bruker D8 advanced powder X-ray diffraction (XRD) system using Cu Kα radiation (λ = 1.5418 Å). The transmission electron microscope (TEM), high-resolution TEM (HRTEM) images and the energy dispersive X-ray spectroscopy (EDS) of the product were characterized using HRTEM (Philips-FEI Tecnai G2S-Twin microscope, Thermo Fisher Scientific, Shangai, China). Field emission scanning electron microscopy (FE-SEM) images were performed by Hitachi SU8010 SEM. X-ray photoelectron spectra (XPS) were measured by ESCALAB 250 XPS spectrometer, Thermo Fisher Scientific, Shangai, China equipped with a monochromic 15 Al Kα X-ray source, Thermo Fisher Scientific, Shangai, China.

Electrochemical performance evaluation: All the electrochemical tests were evaluated in a H-type electrolytic cell divided by Nafion 211 proton exchange membrane with CHI 600E electrochemistry workstation (Shanghai Chenhua Instrument Co. Ltd., Shangai, China). An Ag/AgCl (saturated KCl) as the reference and graphite rod as counter elec-trode. The potentials recorded in this study were transformed into reversible hydrogen electrode (RHE) scale according to formula of E (vs. RHE) = E (vs. Ag/AgCl) + 0.059 pH + 0.197 V. The Nafion 211 membrane can be used only after the following pretreatment. Firstly, it was held in 5% hydrogen peroxide solution at 80 degrees Celsius for 1 h, and then bathed at 80 °C for another 1 h in deionized water. The NRR competence of an elec-trode was assessed using controlled potential electrolysis in a 0.5 M K_2SO_4 electrolyte for 2 h at ambient conditions. The electrolytic cell was purged with N_2 by continuously bub-bled with nitrogen for 30 minutes before the reaction. The electrolyte was frequently bub-bled with N_2 and continue to stir at a stirring rate about ~500 rpm during the electrolysis process.

Determination of NH_3 in 0.5 M K_2SO_4 solution: The concentration of NH_3 produced in 0.5 M K_2SO_4 solutions was confirmed by the indophenol blue method. Firstly, 2 mL of cathodic electrolyte, 2 mL of 1 M NaOH solution including 5% sodium citrate, 5% salicylic acid, 1 mL of 0.05 M NaClO and 0.2 mL of 1% $C_5FeN_6Na_2O\cdot 2H_2O$ were mixed together. Then, UV-Vis absorption spectrum was evaluated at 655 nm before incubating at 25 °C for

1 h. The concentration-absorbance curves were calibrated by standard NH_3 solution with a series of concentrations.

Determination of hydrazine (N_2H_4): The concentration of N_2H_4 in electrolyte was confirmed by the technology of Watt and Chrisp. A mixture of 5.99 g $C_9H_{11}NO$, 30 mL hydrogen chloride and 300 mL ethanol serve as a color reagent. The standard curve was obtained as follows: Firstly, configuring a battery of different concentrations of hydrazine hydrate solution as reference solution by using 0.5 M K_2SO_4; Secondly, 5 mL color reagent was added to 5 mL reference solution and stirring 10 min at 25 °C. Thirdly, the absorption spectra of the mixed solution was presented at 460 nm.

Faradic efficiency and NH_3 yield rate: The Faradic efficiency for NRR was calculated as the amount of electric charge used for compounding ammonia and hydrazine hydrate was divided by the total charge across the electrodes in the electrochemical test. Faradic efficiency = $(3F \times c_{NH_3} \times V)/(17 \times Q)$, where F is the Faraday constant, c_{NH_3} is the concentration of the produced NH_3, V is the number of the electrolyte and Q is the total electric charge for one electron. Meanwhile, the NH_3 yield (y_{NH_3}) is calculated by $y_{NH_3} = c_{NH_3} \times V$. The values of the NH_3 yield rate (R_{NH_3}) can be derived from the slopes of the curves made by plotting the NH_3 yields vs reaction times.

2. Supplementary Figures

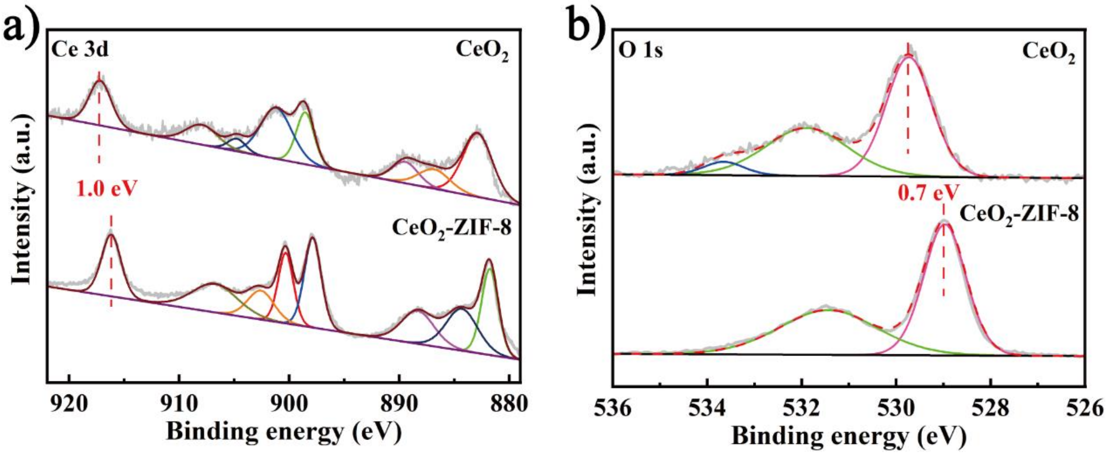

Figure S1. Constitutional element XPS spectra of **a**) Ce 3d and **b**) O 1s of CeO_2 and CeO_2-ZIF-8.

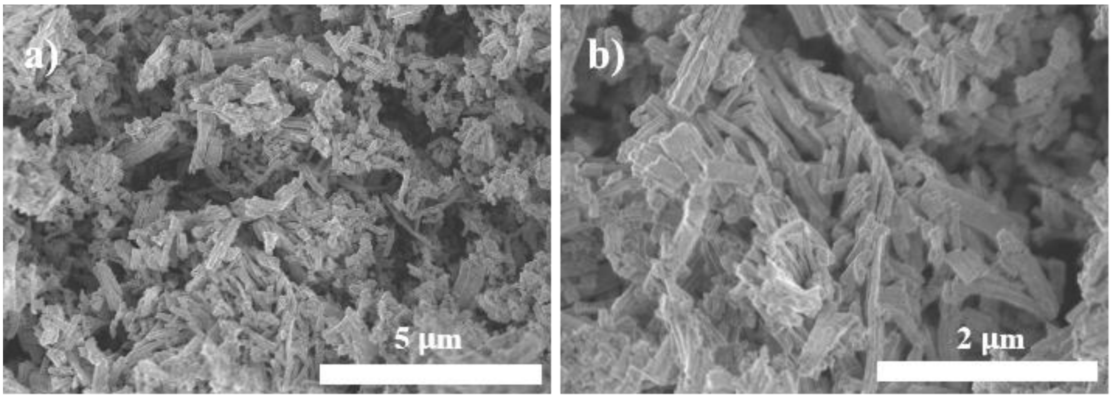

Figure S2. (**a**,**b**) SEM images of CeO_2 with different magnification.

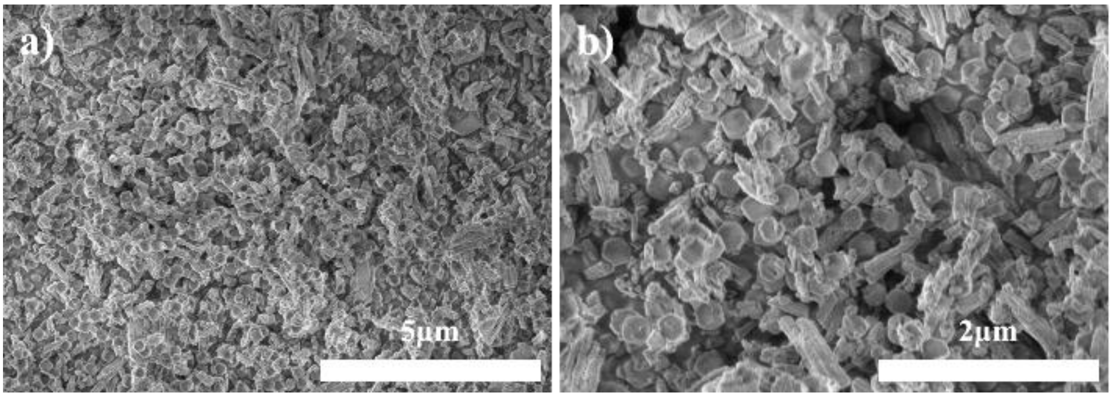

Figure S3. (**a**,**b**)SEM images of CeO_2-ZIF-8 with different magnification.

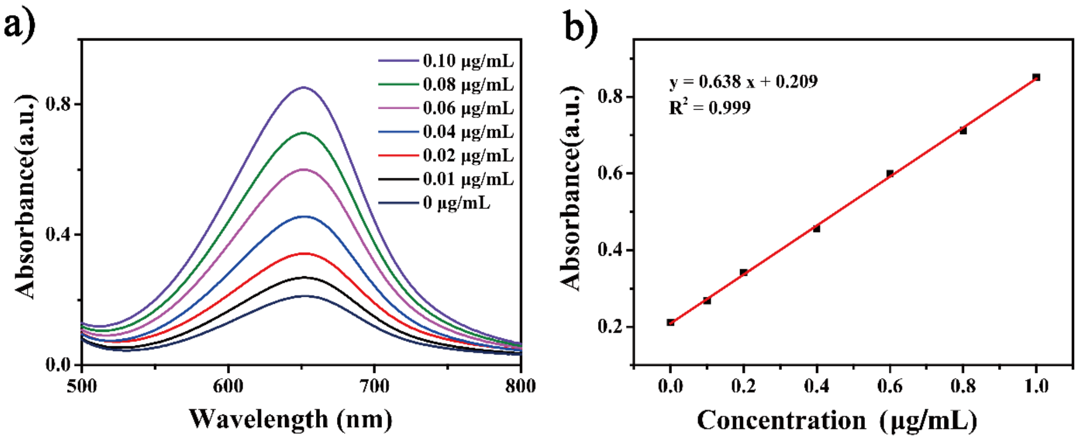

Figure S4. Absolute calibration of the indophenol blue method using ammonium chloride solutions of known concentration as standards. **a**) UV-Vis curves of indophenol assays with NH_4^+ ions after incubated for 2 h and **b**) calibration curve used for estimation of NH_3 by NH_4^+ ion concentration. The absorbance at 655 nm was measured by UV-Vis spectrophotometer, and the fitting curve shows good linear relation of absorbance with NH_4^+ ion concentration ($y = 0.638x + 0.209$, $R^2 = 0.999$) of three times independent calibration curves.

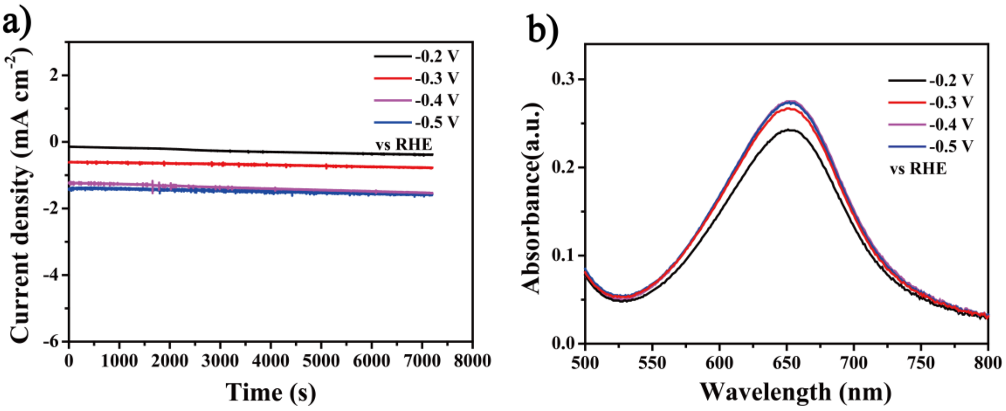

Figure S5. **a**) Chronoamperometry results at the corresponding potentials of CeO_2-ZIF-8/CPs, and **b**) UV-Vis absorption spectra of the K_2SO_4 electrolyte stained with indophenol indicator after charging at each restricted potential vs RHE under N_2 controls for 2 hours.

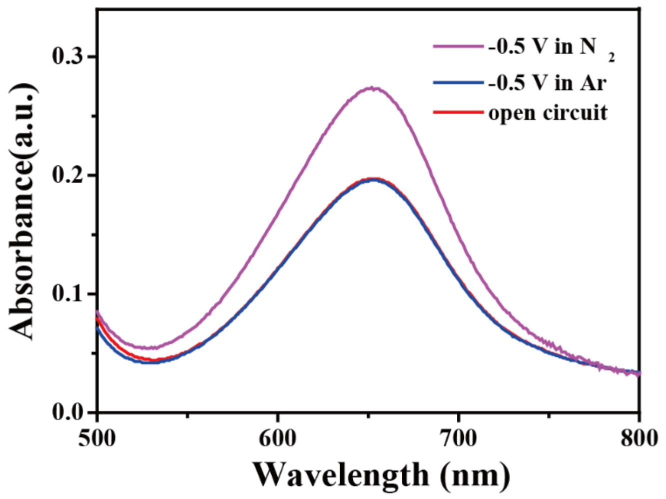

Figure S6. UV-Vis absorption spectra of the electrolytes stained with indophenol indicator after 2 h NRR electrolysis under different conditions: open circuit in N_2 (red curve), -0.5 V in N_2 (purple curve) and -0.5 V in Ar (blue curve).

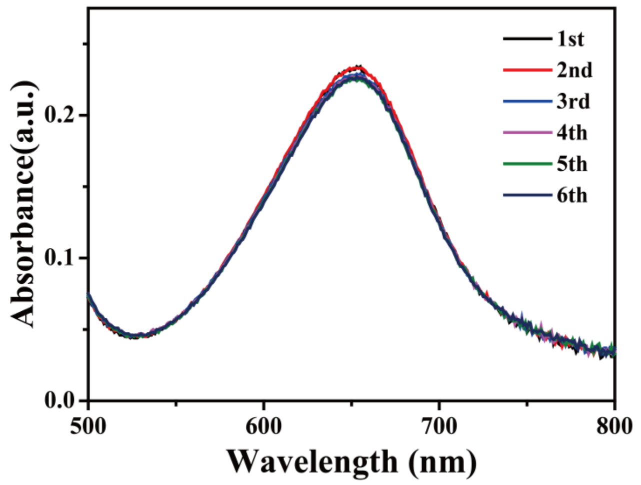

Figure S7. Cycling test of the CeO_2-ZIF-8/CPs after consecutive recycling electrolysis in N_2-saturated 0.5 M K_2SO_4 solution (pH 3.5) at -0.5 V vs RHE for 2 h of each NRR experiment.

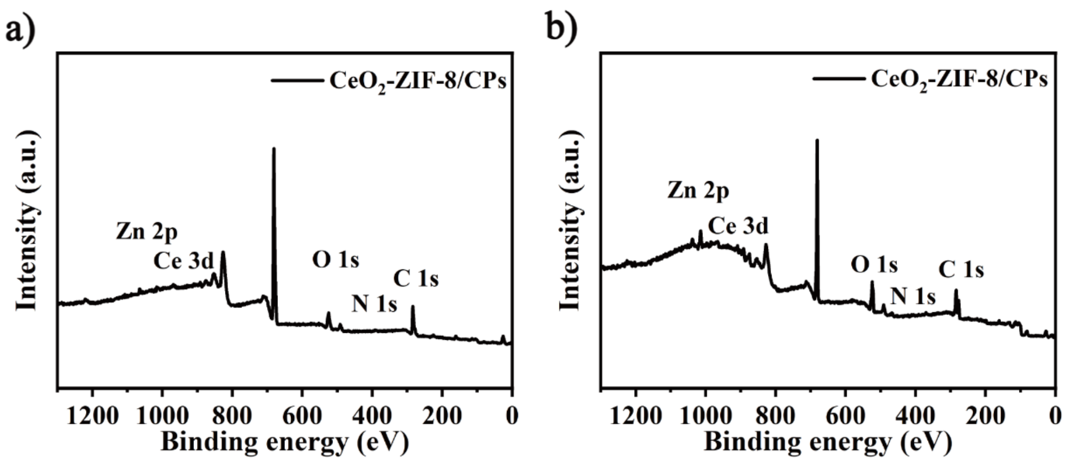

Figure S8. Full XPS spectrum of CeO_2-ZIF-8/CPs before **a**) and after **b**) NRR.

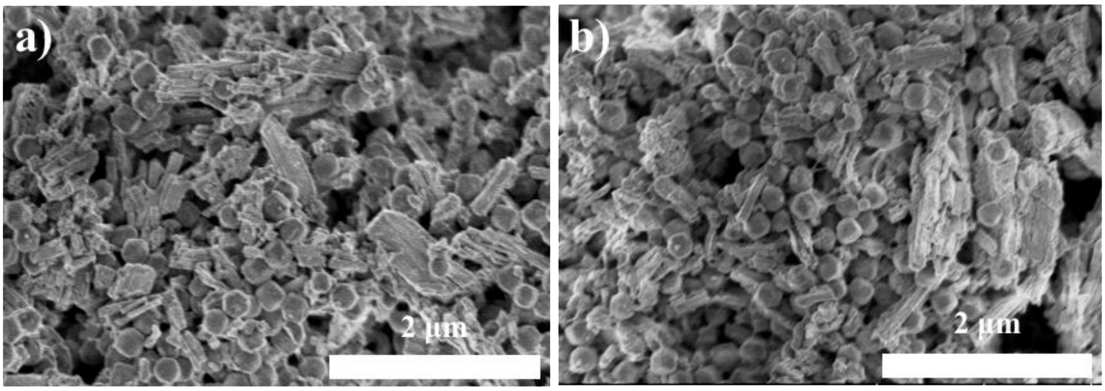

Figure S9. SEM images of CeO_2-ZIF-8/CPs before **a**) and after **b**) NRR.

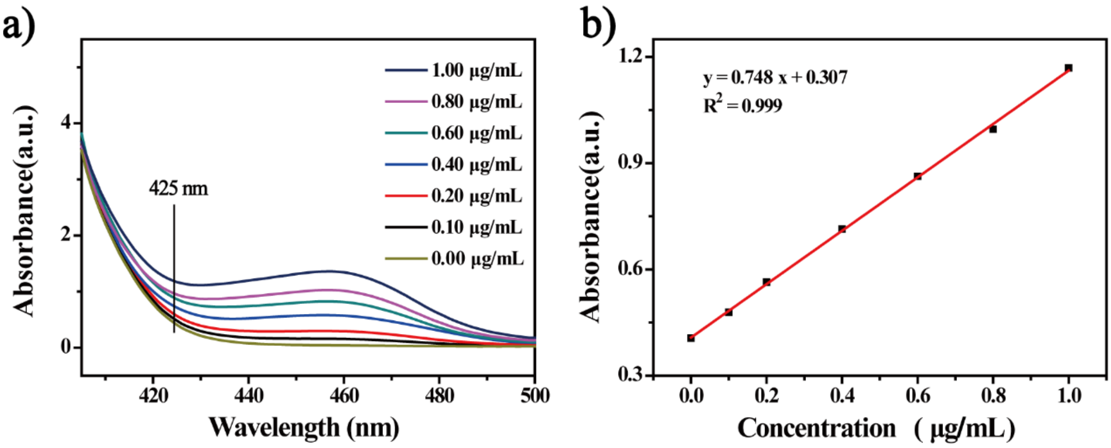

Figure S10. a) UV-Vis absorption spectra of various N_2H_4 concentrations after incubated for 10 min at room temperature. Calibration curve used for estimation of N_2H_4 concentrations. **b**) The absorbance at 425 nm was measured by UV-Vis spectrophotometer, and the fitting curve shows good linear relation of absorbance with $N_2H_4 \cdot H_2O$ concentration (y = 0.748x + 0.307, R^2 = 0.999) of three times independent calibration curves.

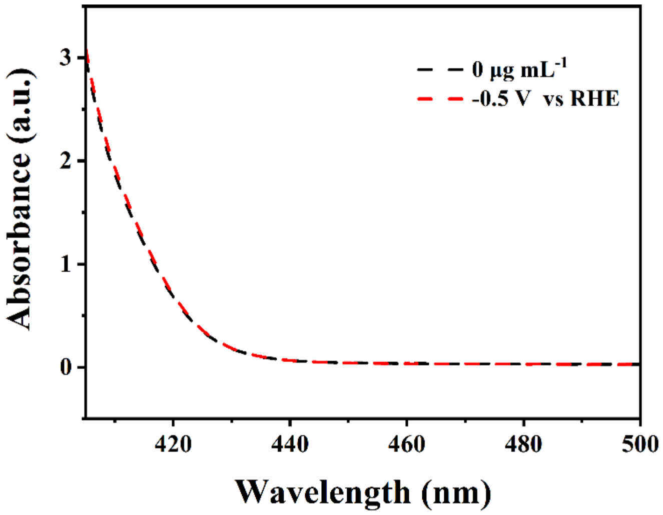

Figure S11. UV-Vis absorption spectrum of the by-produced N_2H_4 for CeO_2-ZIF-8/CPs tested in 0.5 M K_2SO_4 with bubbled N_2 under -0.5 V vs RHE for 2 h.
